# The role for nitric oxide on the effects of hydroalcoholic extract of *Achillea wilhelmsii* on seizure

**Published:** 2014

**Authors:** Mahmoud Hosseini, Fatemeh Harandizadeh, Saeed Niazmand, Mohammad Soukhtanloo, Azadeh Faizpour, Marzieh Ghasemabady

**Affiliations:** 1 *Neurocognitive Research Center, School of Medicine, Mashhad University of Medical Sciences, I. R. Iran*; 2 *Neurogenic Inflammation Research Center, School of Medicine, Mashhad University of Medical Sciences, I. R. Iran*; 3 *Department of Physiology, School of Medicine, Mashhad University of Medical Sciences Mashhad, I. R. Iran*; 4 *Department of Biochemistry, School of Medicine, Mashhad University of Medical Sciences, I. R. Iran*

**Keywords:** *Achillea wilhelmsii*, *Hippocampus*, *Nitric oxide*, *Pentylenetetrazole*, *Rat*, *Seizures*

## Abstract

**Objective** **:** Nitric oxide (NO) plays an important role both as a consequence and as a cause of epileptic seizures. Regarding the central nervous system depressant effects of *Achillea wilhelmsii* *(A. wilhelmsii)*, as well the effects of the plant on NO, this study was aimed to elucidate the possible role for nitric oxide on the effects of hydroalcoholic extract of *A. wilhelmsii* on pentylenetetrazole (PTZ)-induced seizures.

**Materials and Methods**: Fifty-six male Wistar rats were divided into 7 groups (n=8 in each group) and treated with (1) normal saline, (2) normal saline before pentylenetetrazole (PTZ, 90 mg/kg), (3-7) *A. wilhelmsii* extract (100, 200, 400, 800, and 1200 mg/kg) before PTZ. Latency to first minimal colonic seizure (MCS) and the first generalized tonic-clonic seizures (GTCS) as well as the mortality rate were recorded. The brain tissues were then removed for biochemical measurements. Fisher’s exact probability test as well as analysis of variance (ANOVA), followed by Tukey’s test were used for statistical evaluation.

**Results:** Treatment with 100- 1200 mg/kg of the extract did not affect MCS latencies. 400 mg/kg of the extract prolonged GTCS latency (p<0.001), however, the lower and higher doses were not effective. Nitric oxide metabolites concentrations in the hippocampal tissues of the animals treated with 100, 200, and 400 mg/kg of the extract were increased compared with saline (p<0.05-p<0.01).

**Conclusion:** The present study showed that hydroalcoholic extract of *A. wilhelmsii* affects NO metabolites in brain tissues as well the severity of seizures in PTZ-induced seizure model.

## Introduction

Recurring seizures or convulsions are important neurological manifestations of a brain disorder, epilepsy, which afflict about 0.5–1% of the people in the world (Dhir et al., 2006 ▶; Hachinski, 1998 ▶). One of the most commonly used means to study seizure, is administration of pentylenetetrazole (PTZ) to rats or mice which its epileptic effects appear in high doses (more than 40 mg/kg) (Itoh et al., 2004 ▶, Jiang et al., 2004 ▶; Klioueva et al., 2001 ▶). 

These effects consist of two types of motor seizures: 1) minimal and 2) major. The latter is generalized tonic-clonic responses with muscle contractions of the whole body often followed by a cramped tonic state. However, the first one is restricted to forelimbs, and mostly clonic (Klioueva et al., 2001 ▶). 

The cause of increased seizure susceptibility has long been known to be an irregularity in neurotransmitter release in the brain (Coitinho et al., 2001 ▶) or an imbalance in excitatory and inhibitory functions (Peeters et al., 1989 ▶). There are plenty of genetic studies proving that mutation in voltage-gated sodium and potassium channels and nicotinic acetylcholine receptors can be possible responsible mechanisms in this disorder as well as disruption of gamma-aminobutyric acid-ergic (GABAergic) and glutamatergic systems (Baulac et al., 2001 ▶, Coitinho et al., 2001 ▶; Emanuelli et al., 2000 ▶).

NO (Nitric Oxide) has recently drawn a rather increasing attention to itself as an important cellular signaling molecule involved in many physiological and pathological processes. There are three isozymes of nitric oxide synthase (NOS) in the body (De Luca et al., 2006 ▶; Moezi et al., 2012 ▶) whose activity can result in production of NO from its precursor, arginine (Lesani et al., 2010 ▶), amongst which the imbalance of nNOS (neural NOS) is of great importance in neural disorders. NO functions as a vasodilator through stimulating guanylyl cyclase, increasing cyclic guanosine monophosphate (cGMP) production, and regulating the activity of dopaminergic, glutaminergic, and GABAergic systems (Jayakumar et al., 1999 ▶; Moezi et al., 2012 ▶; Paul and Subramanian, 2002 ▶). Some other studies also suggest its role in hormone secretion (Ceccatelli, 1997 ▶) and cell death in human nervous system (Kamoshima et al., 1997 ▶; Nowicki et al., 1991 ▶). It has also been well documented that this gaseous messenger has an important role in pain and analgesia (Hosseini et al., 2011a; Hosseini et al., 2011b; Karami et al., 2011 ▶). Putting all these together, NO’s probable role in convulsion is inferred. Supporting this claim, several investigations were carried out confirming the anticonvulsive influence of NO in convulsions or seizures induced by C-methyl-D-aspartate (Buisson et al., 1993 ▶), penicillin (Marangoz et al., 1994 ▶), kainic acid (KA) (Penix et al., 1994 ▶), picrotoxin (Jayakumar et al., 1999 ▶; Paul and Subramanian, 2002 ▶), and pentylenetetrazole (Lesani et al., 2010 ▶; Moezi et al., 2012 ▶; Shafaroodi et al., 2012 ▶). Other studies in this field have revealed an enhanced expression of type II nitric oxide synthase mRNA in rat brains as a result of consumption of anticonvulsant drugs (Suzuki et al., 2002 ▶). While these studies confirm the anticonvulsive impact of NO, some others pile up evidence against this theory. 

Some investigators demonstrated that a decline in NO production by application of NOS inhibitors or administration of NO precursors leads to inhibition of convulsions evoked by PTZ (Bashkatova et al., 2003 ▶; De Luca et al., 2006 ▶; Itoh et al., 2004 ▶; Osonoe et al., 1994 ▶). As reported, anticonvulsive and proconvulsive effects of NO vary depending upon several factors such as model of seizures, dose of the substance used for evoking seizure, pretreatment time (De Luca et al., 2006 ▶; Paul and Subramanian, 2002 ▶), brain structure and age of animals (De Luca et al., 2006 ▶), source of nitric oxide, and finally, other neurotransmitter systems involvement (Itoh and Watanabe, 2009 ▶; Moezi et al., 2012 ▶). 

Achillea is a plant belonging to the family of Compositae (Nemeth and Bernath 2008 ▶). Many pharmacological properties have been reported for Achillea genus including antiulcer (Cavalcanti et al., 2006 ▶), hepatoprotective (Yaeesh et al., 2006 ▶), anti-inflammatory (Benedek et al., 2008 ▶), antitumor (Csupor-Loffler et al., 2009 ▶; Tozyo et al., 1994 ▶), antispasmotic (Lemmens-Gruber et al., 2006 ▶; Yaeesh et al., 2006 ▶), and choleretic (Benedek et al., 2006 ▶). *Achillea Wilhelmsii* (A. Wilhelmsii), the most important species of Achillea, grows in some countries such as Iran (Asgary et al., 2000 ▶), Egypt, and Turkey (Azadbakht et al., 2003 ▶). 

A. Wilhelmsii is called "boomadaran" in Iran (Lavander cotton) and is found in many areas of the country (Khan and Rezazadeh, 2010 ▶). It has chemical components including borneol, linalol, caryophyllene, 1,8-Cineol, semithujone, flavonoids (rutin), glycoalkaloids, carvacrol, chrysanthenol acetate, and camphor (Afsharypuor et al., 1996 ▶; Azadbakht et al., 2003 ▶; Javidnia et al., 2004 ▶). Some studies have indicated that Achillea species such as A. santolina (Ardestani and Yazdanparast, 2007 ▶), A. ligustica (Tuberoso et al., 2005 ▶), and A. clavennae (Stojanovic et al., 2005 ▶) have antioxidative activity which can reduce free radicals. Moreover, it has been shown that Achillea contains aromatic bitter substances and tannins which have important effects on the nervous system and neurological diseases such as neurasthenia, epilepsy, and seizures (Azadbakht et al., 2003 ▶; Kabuto et al., 1992 ▶).

Regarding the facts that of NO probably has a role in seizure and considering the possible effects of *A. wilhelmsii* on both seizure and NO, this study aimed to elucidate the possible role for nitric oxide on the effects of hydroalcoholic extract of *A. wilhelmsii* on seizure. 

## Materials and methods

**Animals and grouping**

This experimental research was done according to ethics committee guidelines and all the protocols of animal experiments have been approved by the Institution's Animal Care Committee. In this study, 56 virgin male Wistar rats, 250±20 g were used. The animals were maintained in the animal house under controlled conditions including 12/12 h light and dark cycle, 22-24^o^C temperature and 50% relative humidity with laboratory chow and water provided *ad libitum*. 

The animals were divided into 7 groups randomly (*n*=8 in each group) and were treated with (1) Normal saline, (2) Normal saline before PTZ, (3-7) *Achillea* *wilhelmsii* extract (100, 200, 400, 800, and 1200 mg/kg) before PTZ. After PTZ (Sigma aldrich St. Louis, USA) (90 mg/kg body weight, i.p.) injection, the animals were observed for 60 min and the behavioral responses were recorded (Ebrahimzadeh Bideskan et al., 2011 ▶; Hosseini et al., 2011; Hosseini et al., 2009 ▶). Behavioral responses of the animals to PTZ administration were evaluated using these criteria: latency to first minimal clonic seizure (MCS), incidence of MCS, latency to the first generalized tonic–clonic seizures (GTCS), incidence of GTCS, protection percentage against GTCS, and protection percentage against mortality (Ebrahimzadeh Bideskan et al., 2011 ▶; Hosseini et al., 2011; Hosseini et al., 2009 ▶). The brain tissues were then removed and submitted to biochemical measurements. 

**Extracts preparation**

*A. wilhelmsii* was collected from Nishabour city, Khorasan Razavi Province, Iran and identified by botanists in Ferdowsi University of Mashhad, Iran and a voucher number was deposited (4-2012-142). The plants were then dried at room temperature. To prepare hydroalcoholic extract, 50 g of the chopped and dried aerial parts of plant were soaked in ethanol (50%) for 48 h and paper filter was used to filter the solute after mixing. The solvent of the extracts was then removed to dryness with a rotary vacuum evaporator (Rakhshandah and Hosseini, 2006 ▶). The output of the extract was 9%. The extracts were dissolved in normal saline.

**Biochemical assessment**

After behavioral study, the animals were sacrificed, the hippocampi were removed and dissected on an ice-cold surface and submitted to NO metabolite measurements in the tissue. The Griess reaction was adapted to assay nitrates as previously described (Azizi-Malekabadi et al., 2012 ▶; Sadeghian et al., 2012 ▶ ). Briefly, standard curves for nitrates (Sigma, St. Louis, Missouri, USA) were prepared and samples (50 µl serum and 100 µl tissue suspension) were added to the Griess reagent. The proteins were subsequently precipitated by adding 50 µl of 10% trichloroacetic acid (Sigma). The contents were then vortex-mixed and centrifuged and the supernatants were transferred to a 96-well flat-bottomed microplate. Absorbance was read at 520 nm using a microplate reader and final values were calculated from standard calibration plots (Azizi-Malekabadi et al., 2012 ▶; Hosseini et al., 2010 ▶; Sadeghian et al., 2012 ▶).

**Statistical analysis**

Data expressed as mean±SEM. Fisher’s exact probability test, as well as analysis of variance (ANOVA), followed by Tukey’s test, were used for statistical evaluation. p-values less than 0.05 were considered to be statistically significant.

## Results

All the animals in different treatment groups (except for the control group which did not receive PTZ) showed MCS and GTCS following PTZ administration (90 mg/kg). Treatment by 100-1200 mg/kg of the extract didn’t affect MCS latencies (Table 1). 400 mg/kg of the extract prolonged GTCS latency (p<0.001), however, the lower and higher doses were not effective (Table 1). Mortality rate in the animals treated with 200 and 400 mg/kg of the extract was lower than that of PTZ group (p<0.001). There were no significant differences in mortality rate between treated groups by lower and higher doses of the extract compared with PTZ group.

**Table1:** Latencies to minimal clonic seizures (MCS) and generalized tonic–clonic seizures (GTCS) onsets in PTZ and *Achillea* *wilhelmsii* extract (Ex) -treated animals. The animals were treated with normal saline or extract (100, 200, 400, 800, or 1200 mg/kg) before a single injection (90 mg/kg) of PTZ. ^***^p<0.001 as compared with PTZ group

	**MCS latency** ** (Sec)**	**GTCS latency (Sec)**	**Mortality**
**PTZ**	62.2±3.26	102.36±11.14	8/8
**Ext** ** 100**	54.37±4.53	124.5±8.07	8/8
**Ext 200**	50.5±4.31	134.63±33.27	4/8*
**Ext 400**	58.62±13.65	298.63±46.23***	4/8*
**Ext 800**	63.87±4.99	186.88±43.38	5/8
**Ext 1200**	64.37±4.63	196.13±26.37	5/8

**Figure 1:**
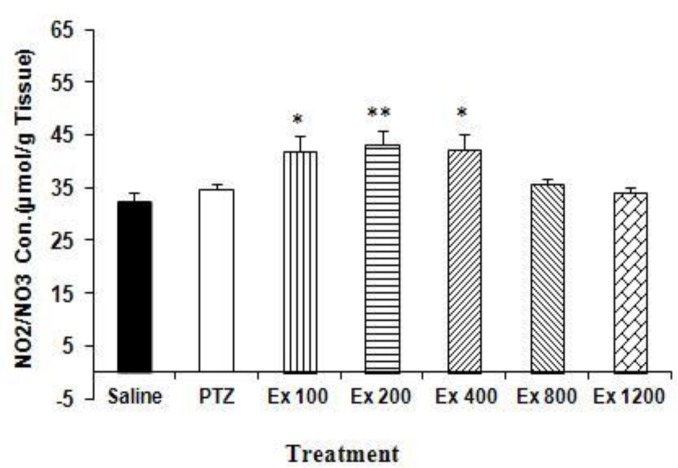
The concentrations of nitric oxide (NO) metabolites (NO2-NO3) in hippocampal tissue in PTZ and *Achillea* *wilhelmsii* extract (Ex) -treated animals. The animals were treated with normal saline or extract (100, 200, 400, 800, or 1200 mg/kg) before a single injection (90 mg/kg) of PTZ.

^*^p<0.05 and ^**^p<0.01 as compared to PTZ group.

There was no significant difference between NO2 or NO3 concentrations in the hippocampal tissues of PTZ-treated group compared with saline. Nitric oxide metabolites concentrations in the hippocampal tissues of the animals treated by 100, 200 and 400 mg/kg of the extract increased compared with sham treated (p<0.05, p<0.01, and p<0.05, respectively). Treatment of the animals with other doses of the extract did not affect the NO metabolites compared with PTZ and saline (Figure. 1). 

## Discussion

In the present study, Nitric oxide metabolites concentrations in the hippocampal tissue of the animals treated with different doses of hydroalcoholic extract *of A. wilhelmsii* were increased compared with normal saline treated ones. In the brain, NO acts as a neuronal messenger and a modulator of neurotransmission (Moncada et al., 1991 ▶). It has been documented that NOS substrates, NO donors, and NOS inhibitors exert various anticonvulsant (Buisson et al., 1993 ▶; Starr and Starr, 1993 ▶) or proconvulsant (Mulsch et al., 1994 ▶, Nidhi et al., 1999 ▶) effects in different seizure models. Bosnak et al. (2007) ▶ showed that systemic administration of L-arginine significantly decreased the frequency of epileptiform electrocorticographical (ECoG) activity on penicillin-induced seizures in male rats while it did not modulate anti-seizures activity of pyridoxine and clonazepam (Bosnak et al., 2007 ▶; Gupta et al., 2000 ▶). However, a proconvulsant activity for L-arginine has also been reported (Mulsch et al., 1994 ▶). Noyan et al.,. (2007) ▶ showed that central administration of L-NAME had no effects on the latency and severity of seizures following pillocarpine injection (Noyan et al., 2007 ▶). It has also been reported that while systemic administration of L-NAME (non-specific NOS inhibitor) had no effects on penicillin-induced seizures in male rats, but 7-nitroindazole (7-NI, a nNOS inhibitor) significantly decreased epileptiform ECoG activity (Bosnak et al., 2007 ▶).

Another research showed that N omega-nitro-L-arginine (NNA), an inhibitor of NOS, aggravated KA-induced seizures (Penix and Davis, 1994 ▶ ). A functional relationship between the NO cGMP signaling pathway and the anticonvulsant activities of adenosine and pyridoxine has also been suggested (Akula et al., 2008 ▶; Bosnak et al., 2007 ▶). The results of our previous study showed that NO has a role in seizures susceptibility following PTZ administration and this effect was different in the presence or absence of ovarian hormones (Hosseini et al., 2009 ▶). In the present study, the concentrations of NO metabolites in hippocampal tissues were not different between convoluted rats by PTZ and control groups.

On the other hand, the animals pretreated with hydroalcoholic extract of *A.* *wilhelmsii* extract showed an elevation in NO metabolites concentrations. It has been reported that *A.* *wilhelmsii* extract has a strong antioxidant activity (Khan and Rezazadeh 2010 ▶; Souri et al., 2010 ▶ ). In contrast to this finding, it was shown that *A. millefolium* administration resulted in a decrease in plasma nitrite and nitrate concentrations in patients with chronic kidney disease (Vahid et al., 2012 ▶). It was also shown that* Achillea santolina* reduced the plasma NO increased in diabetic rats (Yazdanparast et al., 2007 ▶). The extract of Achillea fragrantissima, prevented the nitric oxide overproduction induced by lipopolysaccharide in glial cells (Elmann et al., 2011 ▶ ).

The present study showed that hydroalcoholic extract of *A. wilhelmsii* affects NO metabolites in brain tissues as well the severity of seizures in PTZ-induced seizure model.
